# Mesenchymal Stem Cell Priming: Potential Benefits of Administration of Molecular Hydrogen

**DOI:** 10.3390/ph17040469

**Published:** 2024-04-07

**Authors:** Mikhail Yu. Artamonov, Tyler W. LeBaron, Felix A. Pyatakovich, Inessa A. Minenko

**Affiliations:** 1 MJA Research and Development, Inc., East Stroudsburg, PA 18301, USA; 2 Department of Kinesiology and Outdoor Recreation, Southern Utah University, Cedar City, UT 84720, USA; 3 Molecular Hydrogen Institute, Enoch, UT 84721, USA

**Keywords:** molecular hydrogen, mesenchymal stem cells, stem cell priming, regenerative medicine, tissue regeneration

## Abstract

Stem cell therapy has emerged as a promising avenue for regenerative medicine, offering the potential to treat a wide range of debilitating diseases and injuries. Among the various types of stem cells, mesenchymal stem cells (MSCs) have garnered significant attention due to their unique properties and therapeutic potential. In recent years, researchers have been exploring novel approaches to enhance the effectiveness of MSC-based therapies. One such approach that has gained traction is the priming of MSCs with molecular hydrogen (H_2_). This article delves into the fascinating world of mesenchymal stem cell priming with molecular hydrogen and the potential benefits it holds for regenerative medicine.

## 1. Introduction

**Figure 1:**
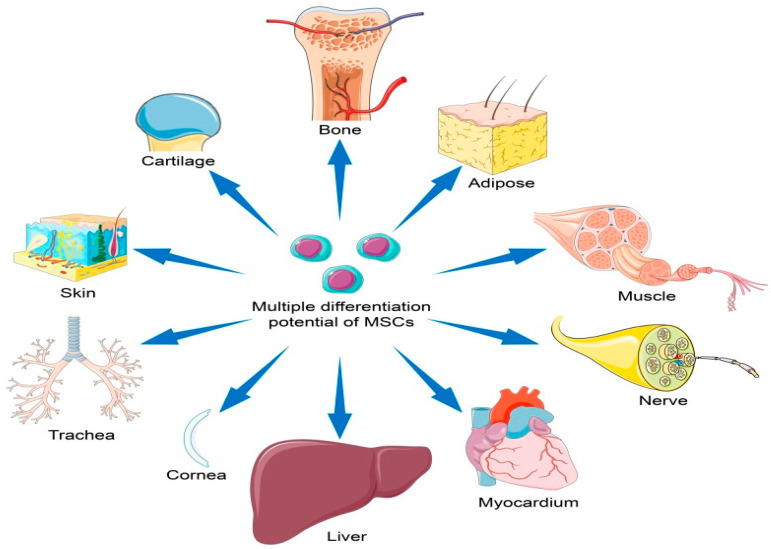
Using mesenchymal stem cells that can differentiate into many different types of cells, tissues can be repaired.

Mesenchymal stem cells (MSCs), also known as multipotent stromal cells, are a type of adult stem cell found in various tissues throughout the body, including bone marrow, adipose tissue, and umbilical cord tissue [1]. MSCs possess the remarkable ability to self-renew and differentiate into different cell types, such as osteoblasts, chondrocytes, and adipocytes [2] (see Figure 1). This plasticity makes them valuable candidates for regenerating damaged tissues and treating diseases characterized by tissue dysfunction. As research continues to uncover their therapeutic potential, MSCs remain a promising avenue for the development of future medical treatments.

MSCs have been extensively studied for their regenerative potential in diverse clinical applications, including in orthopedics, cardiology, neurology, and immunology [3,4]. MSCs are considered safe and effective in treating conditions including Crohn’s disease, systemic lupus erythematosus (SLE), rheumatoid arthritis (RA), GvHD, and Type I diabetes. Searching clinicaltrials.gov for studies using MSCs as an intervention shows 481 studies, which indicates the clinical interest in using MSCs. The reader is referred to recent review articles on the clinical effects and safety of MSCs [5,6,7,8,9,10].

MSCs exert their therapeutic effects through several mechanisms, such as paracrine signaling, immune modulation, and tissue regeneration [11]. Thus, in the therapeutic use of stem cell treatment, MSCs have demonstrated positive outcomes. However, realizing the full regenerative potential of MSCs demands innovative strategies to augment the efficacy of MSC-based therapies. One particularly intriguing and promising avenue in this pursuit is the process of priming MSCs with molecular hydrogen (H_2_). This article explores the ability of molecular hydrogen to prime MSCs.

Sim M. and colleagues report that H_2_ inhalation increases the antioxidant and anti-inflammatory responses in healthy adults [12]. The usage of H_2_ has been found to have an impact on mesenchymal stem cells in experiments using an aplastic anemia model because it increases the number of colony-forming units in these cells [13]. In addition, H_2_ quickly penetrates several organelles, as well as the blood–brain barrier, and is non-toxic even at high concentrations. Moreover, it is used frequently to treat disorders linked to oxidative stress because of its ability to interfere with reactive oxygen species (ROS) in living systems [14]. On the other hand, some researchers note that H_2_ decreases the delayed-onset muscle soreness after downhill running [14]. Some researchers have demonstrated that it improves the muscle function during exercise without having any negative impact on blood oxidative markers [15]. These findings clearly indicate that further research is required to understand how H_2_ performs in a healthy environment. 

Finally, molecular hydrogen has a high safety profile that has been extensively investigated since the 1960s when hydrogen was used to prevent decompression sickness during deep sea diving. Additionally, many pre-clinical and clinical studies have demonstrated that hydrogen is void of toxic effects as has been extensively reviewed previously [16,17]. 

The therapeutic effects of molecular hydrogen, including its antioxidant, anti-inflammatory, and anti-apoptotic effects, uniquely qualify the use of hydrogen to optimize MSCs therapies. For example, MSCs that undergo oxidative stress and aberrant cell signaling and mitochondrial dysfunction will not have the same regenerative and therapeutic effects. The use of H_2_ gas to protect, benefit, and prime MSCs is expected to help overcome the limitations and shortcomings of MSC therapies. As reviewed, there are no indications from studies or hypothetical rationale that there are any contraindications for combining these two therapies. 

Along with other cells, bone marrow contains mesenchymal stem cells. Bone marrow damage is regarded as significant when it occurs throughout the progression of aplastic anemia [18]. Cytokines have significant effects on aplastic anemia. Nonetheless, studies have shown that the cytokines interferon gamma (INF-γ), tumor necrosis factor-alpha (TNF-α), and interleukin-6 (IL-6) are inhibited in the in vivo environment by hydrogen-rich solutions [18]. Meanwhile, colonic bacteria in our bodies continuously create hydrogen, which has no negative consequences on human health [19]. This gives us some information on why H_2_ is a good drug for aplastic anemia [18]. Bacteroides and Firmicutes (the most widespread hydrogen-producing bacteria) are known for their beneficial impacts on human health [20].

Research [21] posits that MSCs, which are uncommon in adult bone marrow, must be expanded ex vivo upon harvesting. However, it has been discovered that such manipulation causes cellular senescence, which causes MSCs to lose their ability to differentiate, proliferate, and have therapeutic potential [22]. Both in vivo and in vitro, oxidative stress is recognized as a major factor leading to cell senescence. However, a promising method to stop the senescence process during MSC growth is hydrogen gas (H_2_) treatment. Notably, it has been shown that exposure to 3% hydrogen gas does not lead to a reduction in the levels of protein carbonyls, hydroxyl radicals, or 8-hydroxydeoxyguanosine, indicating that the effects of 3% hydrogen gas may not be caused by scavenging hydroxyl radicals [21]. H_2_ could reduce the increase in the permeability of the cell membrane, and block the abnormal oxidation of phospholipids, thus protecting cells against cell death [23]. Thus, another crucial mechanism of H_2_ could be blocking lipid peroxidation [23].

The ability of tissues and organs to regenerate can be impacted by senescence. In bone marrow-derived stem cells (BMSCs) treated in vivo with a hydrogen-rich saline solution, the research has shown a reduction in the number of cells positive for senescence-associated galactosidase (SA-Gal) [24]. Rats receiving a hydrogen-rich saline solution (HRS) have BMSCs that are more capable of proliferating, have a greater potential for tri-lineage differentiation, and have fewer cells at the G1 cell cycle arrest than control cells. Additionally, HRS treatment reduces the expression of the senescence-related proteins p53 and p21, as well as the generation of intracellular reactive oxygen species (ROS). Thus, hydrogen can slow down cellular aging in vivo. Hydrogen’s antisenescence actions in BMSCs are mostly mediated by the ROS/p53/p21 signaling pathway [24].

**Figure 2:**
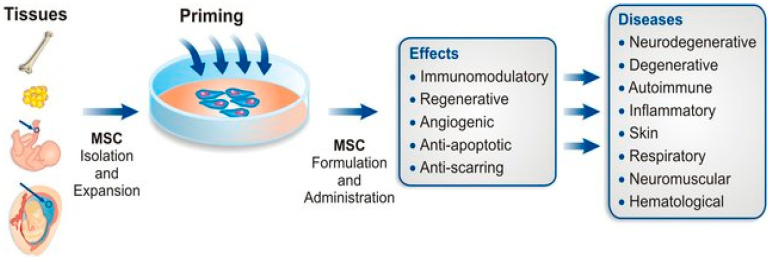
Overview of the production of primed MSCs for the treatment of different disease types. The six steps for primed MSC production are as follows: tissue source selection, MSC isolation, MSC priming (the four main classes of priming approaches that are currently available are represented), MSC expansion, MSC product formulation, MSC administration, and application in different disease types. The rationale is to use different MSC sources/priming approaches for different clinical applications.

The concept of priming MSCs with molecular hydrogen (H_2_) introduces an exciting dimension to their therapeutic potential. Figure 2 illustrates how the administration of molecular hydrogen to MSCs results in the augmentation of the inherent regenerative and immunomodulatory capabilities of these cells. For example, H_2_ dissolved in cell media with isolated stem cells can result in various modulatory effects on the stem cells that improves their therapeutic effects in a wide range of diseases. 

However, despite these favorable effects of using molecular hydrogen to prime MSCs, there are also a number of challenges and difficulties that need to be overcome. For example, since molecular hydrogen is a volatile gas, it does not stay dissolved in solution for an extended period of time and has a half-life of around 2 h. Additionally, since molecular hydrogen is an flammable and explosive gas, many clinics may not have easy access to a tank of the gas due to various regulations and restrictions. Moreover, dissolving the gas into the media may be difficult as it is generally performed under high pressure, which requires special equipment. If the gas is simply bubbled into the media, then it may remove the dissolved O_2_ gas via sparging, which could influence the outcome. Finally, measuring the concentration of H_2_ to ensure that it is at the optimal level (also unknown) can also be a challenge for most clinical settings. Nevertheless, these obstacles are merely challenges that can be overcome with more pre-clinical and clinical research. 

Mesenchymal stem/stromal cell (MSC)-based therapy is promising for the treatment of systemic rheumatic diseases because of the immunomodulatory and regenerative properties of MSCs [13]. The challenges include efficacy, standardization, characterization, safety, as well as MSC outsourcing issues.

## 2. A Historical Journey: The Convergence of Molecular Hydrogen Therapy and Mesenchymal Stem Cell Priming for Regenerative Medicine

The exploration of priming MSCs with molecular hydrogen (H_2_) is a relatively recent development within the broader context of stem cell research and hydrogen therapy. Mesenchymal stem cells, also known as multipotent stromal cells, were first discovered in the mid-20th century. Initially, their role was primarily associated with hematopoiesis, but it soon became evident that MSCs possessed remarkable regenerative potential due to their ability to self-renew and differentiate into diverse cell types, including osteoblasts, chondrocytes, and adipocytes [25].

In parallel, molecular hydrogen therapy emerged in the early 2000s as a novel medical approach. Researchers were drawn to the therapeutic potential of hydrogen gas due to its antioxidant and anti-inflammatory properties [26]. Studies suggested that hydrogen could effectively counteract oxidative stress and inflammation, both of which play critical roles in numerous diseases and injuries.

The concept of priming MSCs with molecular hydrogen started to take shape in the mid-2010s as researchers sought innovative ways to enhance the efficacy of MSC-based regenerative therapies. The hypothesis was that exposing MSCs to hydrogen gas might augment their regenerative capabilities, thereby improving their potential to treat a wide range of debilitating conditions [27]. 

To understand the potential benefits of hydrogen-primed MSCs, researchers initiated initial in vitro and preclinical studies. These investigations aimed to uncover the underlying molecular mechanisms governing the effects of hydrogen gas on MSCs. Specifically, researchers delved into how hydrogen priming influenced MSC differentiation, their secretion of bioactive molecules, and their interactions with the microenvironment [28].

In recent years, there has been a significant translation of research findings into clinical trials and experimental treatments involving hydrogen-primed MSCs. Clinical trials have been launched to rigorously assess the safety and efficacy of hydrogen-primed MSC therapies across various medical fields, including orthopedics, cardiology, neurology, and immunology [29]. These clinical trials represent a crucial step in advancing the practical application of hydrogen-primed MSCs in real-world medical settings.

As the field continues to evolve, researchers are focused on refining priming protocols, optimizing the conditions for hydrogen exposure, and investigating the long-term effects of these therapies. The aim is to fully harness the potential of hydrogen-primed MSCs for the benefit of patients afflicted by a diverse array of diseases and injuries [30,31].

Although this article is primarily focused on the effects of hydrogen on MSCs, it should be noted that there is also a growing number of studies on using hydrogen on other stem cell types. For example, a recent 2023 study demonstrated that molecular hydrogen treatment activated epidermal stem cells and accelerated wound healing [30]. Administering a high concentration of H_2_ (66% H_2_) in a cutaneous aseptic wound model resulted in a healing rate three times higher than the control group on day 11 post-wounding. Notably, the study employed advanced analyses, highlighting the significant impact of H_2_ on fast re-epithelialization, early deposition of extracellular matrix (ECM) components, and the activation of epidermal stem cells. This novel pattern of wound healing induced by H_2_ treatment offers promising insights into its potential influence on stem cell behavior, suggesting positive effects on cell viability and mitochondrial functions across various cell types.

Another recent 2023 study demonstrated that molecular hydrogen plays a crucial role in promoting myogenic differentiation in adipose-derived stem cells (ADSCs) for potential therapeutic applications in skeletal muscle injuries [32]. The study investigated the protective effects of H_2_ on ADSCs using various assays, including the MTT assay, live–dead cell staining, Western blot analysis, immunofluorescence staining, confocal imaging, and transmission electron microscopy. The results indicated that an appropriate volume fraction of H_2_ significantly reduced mitochondrial reactive oxygen species levels, increased the number of mitochondria, and induced mitophagy. These effects collectively enhanced the survival and myogenic differentiation of ADSCs [32]. These findings suggest the promising application of H_2_ in addressing skeletal muscle diseases and pathologies associated with mitochondrial dysfunction.

These and other studies illustrate similar patterns of molecular hydrogen effects on MSCs and MSC-derived therapies since the mechanisms of action (discussed below) are also similar. The ongoing progress in this field holds the promise of revolutionizing regenerative medicine and providing new avenues for addressing challenging medical conditions. 

However, it is imperative to emphasize that this journey is still in its nascent stages, and there is much to learn and discover. Research and clinical evaluation must persist to confirm the safety, efficacy, and long-term effects of hydrogen-primed MSC therapies. With each milestone reached and each question answered, we move closer to realizing the full potential of this remarkable convergence and the transformative possibilities it holds for the future of regenerative medicine [33]. 

## 3. Mechanisms of MSC Priming using Molecular Hydrogen

Before delving into the mechanisms of MSC priming using molecular hydrogen, it is essential to understand the context of this innovative approach. Mesenchymal stem cells (MSCs) have long held promise in regenerative medicine and immunotherapy due to their ability to differentiate into various cell types and modulate immune responses. However, to harness their full therapeutic potential, researchers have explored methods to enhance their survival, immunomodulatory capabilities, and tissue regenerative properties. One such method involves priming MSCs with molecular hydrogen (H_2_), a selective antioxidant and anti-inflammatory agent. This priming process aims to equip MSCs with additional tools to combat oxidative stress, reduce inflammation, and improve their overall therapeutic effectiveness. Now, let us delve into the intricate mechanisms through which molecular hydrogen enhances MSCs.

The influence of H_2_ on human physiology has been summarized into four main molecular bases:

(1) A precise counteracting action against ^•^OH;
(2) Mitigating the action of ONOO^−^;
(3) Signal modulation;
(4) Modification of gene expression.

The initial mechanism recognized for H_2_ was its precise scavenging of hydroxyl radicals and mitigation of other ROS. Undeniably, biomarkers of oxidative stress such as 8-hydroxydeoguanosine (8-OHdG) (nucleic acid oxidation marker), 4-hydroxyl-2-nonenal (4-HNE) (a specific marker of lipid peroxidation), and malondialdehyde (MDA) are reduced in all the surveyed rat models [34].

**Figure 3:**
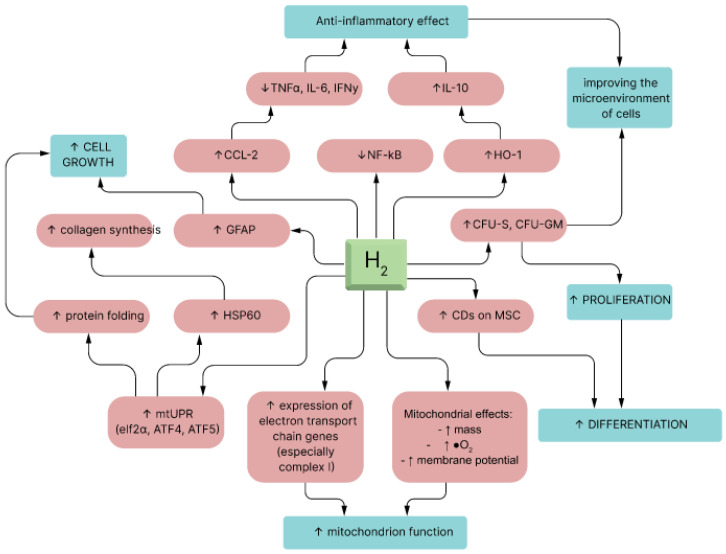
Molecules that are elevated by H_2_ administration [42].

The other molecular mechanism of H_2_’s influence is a ONOO^−^ neutralizing action [35]. Although H_2_ could not eradicate ONOO^−^ as effectively as ^•^OH in vitro, H_2_ could capably decrease ONOO^−^-stimulated generation of nitrotyrosine [36,37]. Similarly, the ONOO^−^ molecule exerts therapeutic properties, such as dilating blood vessels and suppressing platelet accumulation [38,39,40]. These mechanisms are not mutually exclusive and some of them could be reasonably related to other mechanisms. Nevertheless, additional studies are essential to explain the detailed relationships between these mechanisms [35]. Earlier studies were repeatedly concentrated on the first two mechanisms, and limited research has been conducted on gene expression and modulating signaling pathways [41] (see Figure 3).

### 3.1. Reduction of Oxidative Stress

Molecular hydrogen (H_2_) emerges as a potentially useful agent in the context of MSC priming due to its ability to mitigate oxidative stress. Oxidative stress, a physiological imbalance between reactive oxygen species (ROS) and antioxidants, plays a pivotal role in cellular damage and dysfunction. Among the harmful ROS species, hydroxyl radicals and peroxynitrite are particularly notorious for their detrimental effects on cellular components, including lipids, proteins, and DNA.

The uniqueness of H_2_ as a selective antioxidant lies in its ability to specifically target and neutralize these highly reactive ROS, sparing essential cellular components from oxidative damage [43]. When MSCs are primed with H_2_, they receive an invaluable shield against the oxidative onslaught that often accompanies transplantation procedures or the exposure to inflammatory microenvironments within the body. This preservation of cellular integrity and functionality is pivotal for ensuring the success of MSC-based therapies.

In the priming process, H_2_ acts as a guardian, reinforcing the resilience of MSCs. It safeguards the structural and functional integrity of these multipotent stromal cells, thus enhancing their therapeutic potential [44]. This protection against oxidative stress not only extends the lifespan of MSCs but also ensures their continued ability to effectively secrete trophic factors, modulate immune responses, and promote tissue regeneration.

By reducing oxidative stress, H_2_ empowers primed MSCs to thrive in the challenging environments they encounter within the host’s body, ultimately translating into more robust and durable therapeutic outcomes. This aspect of molecular hydrogen priming underscores its significance in harnessing the full regenerative potential of MSCs, fostering optimism for the future of regenerative medicine.

### 3.2. Anti-Inflammatory Effects

In the realm of regenerative medicine, one of the pivotal aspects of molecular hydrogen (H_2_) priming of MSCs lies in its anti-inflammatory properties. Inflammation is a complex and essential part of the body’s response to injury or infection. However, when it becomes chronic or excessive, inflammation can contribute to tissue damage and the progression of various diseases. The ability of H_2_ to modulate inflammatory pathways and enhance MSCs’ anti-inflammatory potential holds significant promise for a wide range of medical conditions characterized by inflammatory dysregulation [45].

H_2_’s anti-inflammatory effects stem from its ability to influence various signaling pathways involved in the inflammatory response. One key facet of this modulation is the downregulation of pro-inflammatory cytokines and chemokines, including tumor necrosis factor-alpha (TNF-α) and interleukin-6 (IL-6). These molecules are central players in the inflammatory cascade, orchestrating the recruitment of immune cells and the amplification of inflammation.

When MSCs are primed with H_2_, their inherent ability to suppress inflammation is further amplified and refined. These primed MSCs possess a heightened capacity to regulate the immune response, serving as natural dampeners of excessive inflammation [45]. This augmentation is particularly valuable in clinical scenarios where uncontrolled inflammation is a driving force behind tissue damage, as seen in autoimmune diseases, acute injuries, and chronic inflammatory disorders.

The priming of MSCs with molecular hydrogen essentially equips these cells with enhanced anti-inflammatory weaponry. This process unfolds as follows:

**a**  **Augmented Immune Modulation:**
  MSCs in their native state already exhibit a remarkable ability to modulate the immune system. They can interact with immune cells, such as T cells and macrophages, to promote an anti-inflammatory environment. When primed with H_2_, these interactions become more potent and finely tuned [46]. Primed MSCs can more effectively suppress the activation of pro-inflammatory immune cells while promoting the expansion of regulatory immune cell populations, ultimately tipping the balance toward immune tolerance and dampening inflammation.
**b**  **Targeting Inflammatory Mediators:**
  H_2_’s influence extends beyond the cellular level to the molecular level. By reducing the expression of pro-inflammatory cytokines like TNF-α and IL-6, H_2_ helps to curtail the propagation of inflammation [47]. These cytokines are known to be major contributors to the cytokine storm observed in conditions like severe acute respiratory syndrome (SARS), a feature often seen in severe cases of viral infections [48]. When primed MSCs enter the inflammatory milieu, they act as localized “firefighters”, effectively dousing the flames of excessive inflammation.

The clinical implications of MSC priming with molecular hydrogen for anti-inflammatory purposes may be profound. Consider autoimmune diseases like rheumatoid arthritis, where the immune system mistakenly attacks the body’s own tissues, leading to chronic inflammation and joint damage [49]. Primed MSCs have the potential to exert more robust control over the inflammatory processes at play, offering relief to patients and potentially slowing the progression of the disease.

In acute injuries such as traumatic brain injuries or spinal cord injuries where inflammation can exacerbate tissue damage, the use of primed MSCs could limit secondary injury cascades and improve recovery outcomes. Additionally, in chronic inflammatory disorders like Crohn’s disease or psoriasis, where patients often struggle with recurring and debilitating symptoms, the enhanced anti-inflammatory capabilities of primed MSCs may offer new avenues for managing and alleviating these conditions. Researchers are continually exploring MSC priming with molecular hydrogen within the context of inflammation [50]. As a result, this has opened up intriguing prospects for future research, as well as clinical applications. 

Furthermore, this avenue of research highlights the versatility and adaptability of MSC-based therapies. By enhancing the inherent anti-inflammatory properties of these cells, priming with molecular hydrogen represents a promising strategy for addressing a wide array of diseases characterized by immune dysregulation and excessive inflammation [49]. As our understanding deepens and the clinical evidence accumulates, the convergence of MSCs and molecular hydrogen priming could become a cornerstone in the management of inflammatory disorders, ushering in a new era of precision medicine and improved patient outcomes.

### 3.3. Preservation of Mitochondrial Function

Oxidative stress can damage mitochondria, the powerhouses of cells, leading to impaired cellular energy production and increased ROS generation. H_2_ can help protect mitochondria by reducing ROS levels [51]. Priming MSCs with H_2_ may enhance their energy-producing capacity, making them more resilient when exposed to the energy demands of tissue repair and regeneration processes. This can be crucial for the success of MSC-based therapies.

### 3.4. Immunomodulation

MSCs have inherent immunomodulatory properties, which allow them to regulate the immune system’s response [45]. They can suppress the activation of immune cells, such as T cells and macrophages, and promote the generation of regulatory T cells (Tregs) [52]. When MSCs are primed with H_2_, this immunomodulatory capacity is further enhanced [53]. H_2_ can influence the expression of molecules involved in immune regulation, thereby improving MSCs’ ability to modulate immune responses [53]. This is especially relevant in conditions like graft-versus-host disease (GVHD) or organ transplantation, where immune dysregulation is a major concern.

### 3.5. Enhanced Engraftment and Survival

Transplanted MSCs often face challenges in terms of engraftment and survival within the host tissue. The hostile microenvironment, which includes inflammation and oxidative stress, can compromise their viability [54]. Priming MSCs with H_2_ can help increase their resistance to these hostile conditions, improving their chances of engraftment and long-term survival. This is particularly important for achieving sustained therapeutic effects in various diseases.

### 3.6. Synergistic Effects

The combination of H_2_’s antioxidant and anti-inflammatory properties with MSCs’ regenerative and immunomodulatory capabilities creates a potential synergistic effect [52]. This synergy can lead to improved therapeutic outcomes in conditions where these mechanisms are involved.

### 3.7. Tailored Priming Strategies

Researchers are exploring various strategies for priming MSCs with H_2_, including pre-conditioning the cells before transplantation or co-administering H_2_ with MSCs during transplantation [55]. The choice of strategy may depend on the specific disease being targeted and the desired therapeutic outcome.

**Figure 4:**
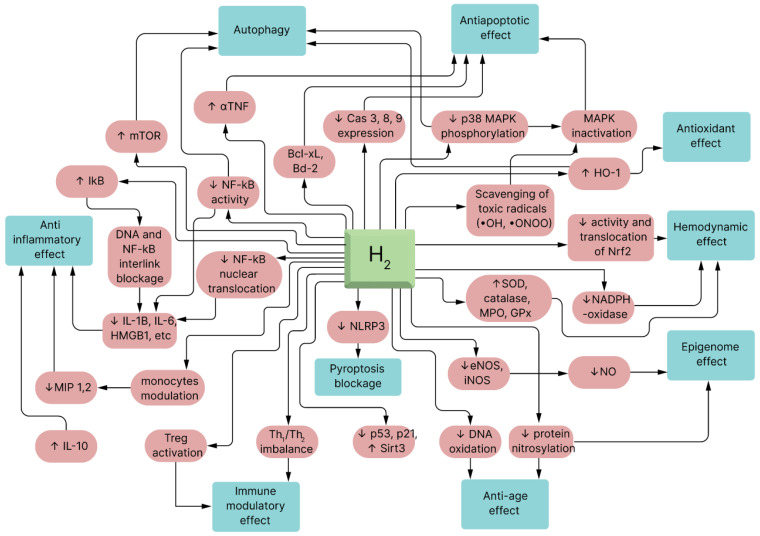
Molecular effects of hydrogen in living organisms. The figure shows some of the proposed mechanisms by which the main effects of hydrogen are mediated on the molecular and cellular levels. The brown links represent shifts in regulatory molecules that lead to the development of specific cellular effects (indicated by green blocks). Abbreviations: MAPK—mitogen-activated protein kinase, HO—heme oxygenase, TNF—tumor necrosis factor, SOD—superoxide dismutase, MPO—myeloperoxidase, NOS—nitric oxide synthase (iNOS—inducible NOS; eNOS—endothelial NOS), GPx—glutathione peroxidase, Cas—caspase, HMGB1—high-mobility group protein B1, NLRP—nucleotide-binding oligomerization domain, Th—T-cytotoxic lymphocyte.

Priming mesenchymal stem cells with molecular hydrogen is a promising approach that capitalizes on H_2_’s antioxidant, anti-inflammatory, and immunomodulatory properties to enhance the therapeutic potential of MSC-based therapies [54]. This strategy has the potential to improve the outcomes of regenerative medicine applications and immunotherapies in a wide range of medical conditions, but further research and clinical trials are needed to fully validate its effectiveness and safety (see Figure 4).

## 4. Methods of Administering Molecular Hydrogen for Stem Cell Priming and for Therapeutic Use

The main method of providing H_2_ gas to prime MSCs has been placing the stem cells in an atmosphere with a specified concentration of molecular hydrogen. After 20–30 min, the H_2_ molecules dissolve into the media and reach an equilibrium, which can be predicted using Henry’s Law. The concentration generally ranges from 2% to 66.67%, with the high end from exposing the MSCs to oxyhydrogen (66.67% H_2_/33.33% O_2_). The duration of exposure may be as short as 60 min or up to several days. This also speaks to the dosing aspect of molecular hydrogen, which remains highly elusive. It is unknown what the optimal concentration, frequency, or duration of molecular hydrogen exposure is [13]. This is a significant hurdle that needs to be overcome by future mechanistic and clinical research. 

Molecular hydrogen (H_2_) can be introduced into the body through a diverse array of methods, each offering unique advantages and characteristics. It is essential to recognize that these approaches not only vary in their convenience for specific medical conditions (for instance, hydrogen baths might be preferable for dermatological ailments) [56] but they can also influence the pharmacokinetics of the molecule, consequently altering its pharmacological effects [56].

Historically, one of the earliest methods for delivering hydrogen was through hyperbaric chambers containing an enriched atmosphere of hydrogen gas. Despite promising outcomes in experiments conducted by Dole et al. in 1975 [57], this approach was not widely adopted, possibly due to practical implementation challenges.

Currently, the most prevalent options for molecular hydrogen therapy include inhaling gas mixtures containing H_2_, consuming hydrogen-saturated water, and administering intravenous or intraperitoneal injections of a sodium chloride solution saturated with H_2_. Each of these delivery routes possesses its own distinct characteristics, advantages, disadvantages, and potential mechanisms of action [58].

Hydrogen therapy studies have primarily employed hydrogen-saturated solutions (such as hydrogen water or hydrogen saline). However, research on hydrogen inhalation, particularly in clinical applications, is on the rise. Inhaling hydrogen is a relatively straightforward method for both laboratory animals and humans, offering precise control over the hydrogen dosage by regulating the exposure time and gas concentration. Nonetheless, it is important to note that molecular hydrogen is flammable and explosive when it reacts with oxygen. Thus, precautions must be taken when using gas mixtures with hydrogen concentrations exceeding 4%. Despite this, higher concentrations, such as 66.67% H_2_ and 33.33% O_2_, have been employed, particularly in treating COVID-19 patients in China [59]. Efficacy in treating conditions like chronic obstructive pulmonary disease and severe bronchial asthma has also been reported with H_2_ inhalation [59]. The apparent dose-dependent nature of hydrogen’s antioxidant and anti-inflammatory properties underscores the relevance of this approach.

Given hydrogen’s physicochemical properties and its small molecular size and weight, hydrogen inhalation offers systemic effects. It readily diffuses through alveolar walls into the bloodstream, reaching various organs and tissues. Studies have demonstrated that the continuous inhalation of a gas mixture containing 2.4% hydrogen for 72 h in healthy animals does not significantly affect physiological parameters [58]. Simultaneously, sanogenetic effects on various organs and tissues have been observed [58].

This approach eliminates the explosion and fire hazards associated with the other methods and ensures portability, making hydrogen water widely accessible. However, this method has limitations due to the relatively low solubility of the gas in water. At standard atmospheric pressure and room temperature, the saturation of dissolved hydrogen is only 0.78 mM (1.57 mg/L). However, much higher concentrations (e.g., >10 mg/L) can be achieved by using pressure [13]. Nevertheless, the relatively limited solubility can sometimes hinder reaching the necessary dosage for an optimal clinical effect. Additionally, hydrogen water should be consumed immediately after preparation because its hydrogen concentration diminishes rapidly. Furthermore, over 90% of ingested hydrogen is lost through exhalation [13], suggesting a high uptake in the gastrointestinal tract and venous circulation, with minimal penetration into brain cells, which has implications for clinical indications.

To address these challenges, alternative methods have been explored, such as creating nanocomposites for delayed gas release. These nanocomposites can be incorporated into oral tablets, ensuring high patient compliance. For example, hybrid palladium nanocrystals have been experimentally tested and showed promise in oncology and protecting cells from hyperthermia-induced oxidative stress [60]. Various elements have been explored as bases for hydrogen-releasing nanocomposites, including silicon particles [60].

Microbubble systems represent another avenue for targeted hydrogen delivery, maximizing bioavailability while minimizing gas loss. This method allows for the administration of higher gas volumes compared to hydrogen-saturated water [60]. Its effectiveness has been demonstrated, particularly in rat ischemic myocardial injury models [60].

The third significant approach involves injections and infusions of isotonic saline solutions saturated with hydrogen [13]. This method enables precise hydrogen dosing, using varying concentrations, which can increase the bioavailability for target organs and produce localized effects on specific tissues when necessary. However, it does come with risks, such as infection, and requires skilled medical personnel for administration. This administration is primarily performed intravenously (in patients) or intraperitoneally (in laboratory animal studies). The clinical potential of this route has yet to be fully explored, similar to intravenous ozone therapy, which also utilizes medical gases [13]. 

**Figure 5:**
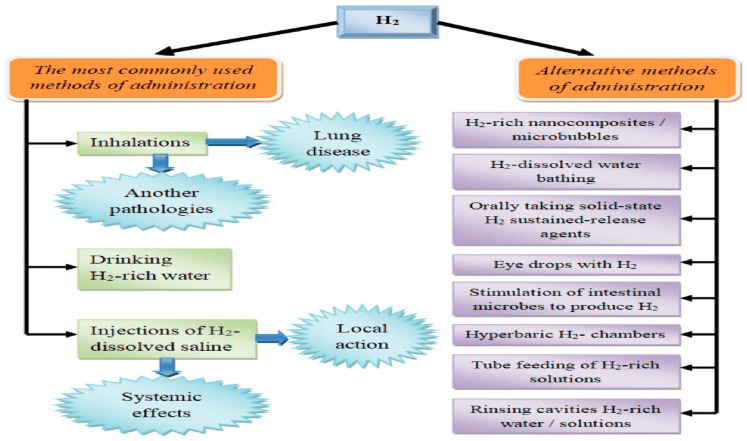
Routes of molecular hydrogen administration.

Various alternative methods for using molecular hydrogen have emerged, offering both systemic and localized effects (Figure 5). For example, hydrogen baths have been effectively used in dermatology and cosmetology for conditions like psoriasis and liposuction [13]. Another application involves using hydrogen-saturated eye drops to treat ischemic iris lesions and suppress apoptosis [13]. Stimulating endogenous hydrogen synthesis through symbiotic microflora is an intriguing avenue, where oral lactulose administration increases hydrogen production in the gastrointestinal tract [13]. A hydrogen breathing test has even been proposed to assess intestinal microflora based on their hydrogen-producing capacity [61]. It is important to note that hydrogen is synthesized alongside other gases, like methane, by microorganisms. The tissue distribution of hydrogen varies when using these routes, with limited penetration into brain cells when hydrogen water is consumed [61], potentially impacting clinical indications.

In conclusion, there is a wide range of approaches to introduce molecular hydrogen into the body, each with its unique attributes, topical characteristics, and effects on hydrogen’s pharmacokinetics. The choice of method depends on factors such as the desired therapeutic outcome, practicality, and safety considerations. Researchers continue to explore these methods to refine their applications and maximize the benefits of molecular hydrogen therapy for diverse medical conditions.

## 5. Expanding the Horizons of Mesenchymal Stem Cell (MSC) Priming with Molecular Hydrogen: Unveiling Key Applications

Before delving into the specific potential applications, it is crucial to emphasize that the priming of MSCs with molecular hydrogen (H_2_) has opened up a new frontier in regenerative medicine and immunotherapy. This innovative approach, combining the remarkable regenerative capabilities of MSCs with the multifaceted properties of H_2_, holds immense promise for addressing a diverse spectrum of medical conditions. By bolstering the survival and functionality of MSCs, reducing oxidative stress, mitigating inflammation, and enhancing immunomodulation, this approach represents a paradigm shift in how to approach various diseases. Now, let us explore in detail the exciting potential applications where H_2_-primed MSCs can make a significant impact on patients’ lives.

MSC priming with molecular hydrogen (H_2_) constitutes a pioneering approach at the intersection of regenerative medicine and immunotherapy, with diverse and profound applications across various medical domains. In the realm of neurodegenerative diseases, H_2_-primed MSCs offer a ray of hope for patients battling conditions like Alzheimer’s and Parkinson’s by providing neuroprotection through the simultaneous reduction of oxidative stress and inflammation [31,62]. Cardiovascular diseases, particularly myocardial infarction, may witness significant advancements in cardiac tissue repair facilitated by H_2_-primed MSCs [63]. The spectrum of potential applications broadens further, extending to autoimmune disorders such as rheumatoid arthritis and multiple sclerosis, where H_2_-primed MSCs display the ability to enhance immunomodulation and stimulate tissue regeneration [64,65]. Furthermore, the therapeutic potential of H_2_-primed MSCs is evident in addressing tissue injuries, spanning from bone and cartilage to skin, promising accelerated and more effective healing [66]. In the realm of respiratory diseases, H_2_-primed MSCs have the potential to mitigate inflammation and facilitate lung tissue repair in conditions like chronic obstructive pulmonary disease (COPD) and acute respiratory distress syndrome (ARDS) [67]. Beyond these, H_2_-primed MSC therapies can be tailored for gastrointestinal disorders, organ transplantation, sports injuries, and musculoskeletal conditions, all of which stand to benefit from the enhanced healing and recovery potential [68,69,70]. Despite the wide array of promising applications, it is imperative to underscore that rigorous research and comprehensive clinical trials are pivotal in validating the safety and efficacy of this approach within each specific medical context [71].

**Figure 6:**
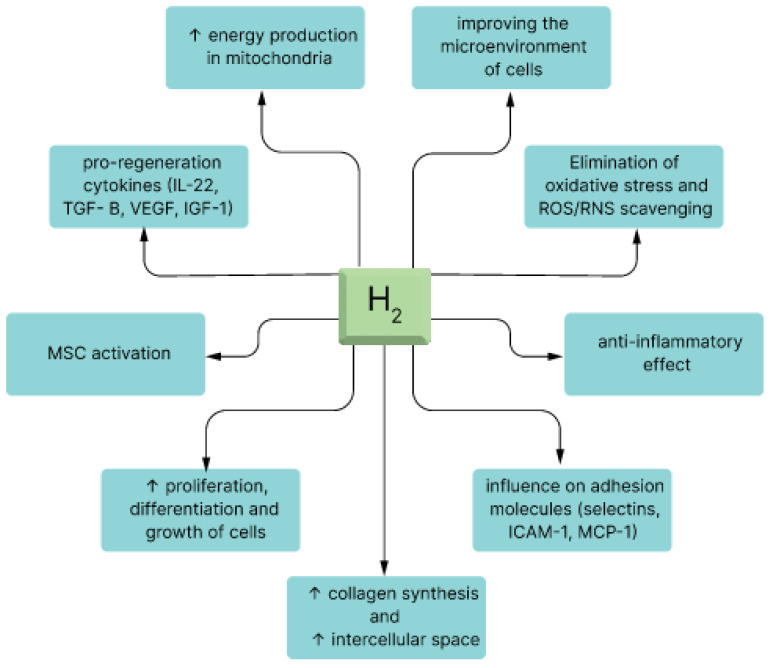
Key applications of molecular hydrogen.

Furthermore, the potential of MSC priming with molecular hydrogen extends beyond its immediate therapeutic applications. It opens exciting possibilities for advancing our understanding of stem cell biology and regenerative medicine. The ongoing research into the molecular mechanisms involved in stem cell biology provides valuable insights that may have broader implications for enhancing the regenerative potential of various stem cell types. As we continue to unravel the intricacies of molecular hydrogen priming, it is conceivable the novel approaches and innovative strategies developed can be applied not only to MSCs but also to other stem cells, pushing the boundaries of regenerative medicine even further and offering new avenues for addressing unmet medical needs (see Figure 6).

## 6. Navigating Complex Challenges and Considerations in the Pursuit of H_2_-Primed Mesenchymal Stem Cell Therapies

In the pursuit of harnessing the therapeutic potential of molecular hydrogen (H_2_) to prime MSCs, several intricacies and challenges emerge, demanding careful consideration. One such challenge lies in determining the optimal dosage—a precise concentration and duration of H_2_ exposure required for effective MSC priming. This crucial task, though pivotal, remains an ongoing endeavor, necessitating continuous research and experimentation to strike the right balance for maximal therapeutic impact [72,73].

Achieving the optimal dosage is a multifaceted challenge. It involves not only defining the appropriate concentration of H_2_ but also considering the duration of exposure. Researchers must take into account factors such as the specific disease or condition being targeted, the age and health status of the patient, and the delivery method for the H_2_. Additionally, variations in individual responses to H_2_ therapy underscore the complexity of this dosage determination process.

Furthermore, paramount importance is placed on exploring the safety and long-term effects of H_2_ administration. While H_2_ is generally regarded as safe, thorough investigations are imperative to discern any potential interactions with concurrent medications or therapies and to establish its sustained safety profile [74,75].

Safety assessments extend beyond short-term exposure, necessitating long-term monitoring to uncover any unforeseen adverse effects or interactions. Considering the potential applications of H_2_-primed MSC therapies across a wide spectrum of medical conditions, comprehensive safety evaluations are essential to ensure patient well-being.

Moreover, transitioning from preclinical research to large-scale clinical trials poses a significant hurdle in clinical translation. Rigorous clinical studies are indispensable for assessing the actual safety, efficacy, and clinical utility of H_2_-primed MSC therapies. This transition from laboratory findings to large-scale clinical trials is pivotal, ensuring that these innovative therapies can be effectively employed to address a wide range of medical conditions, thereby offering new hope to patients around the world [76,77]. 

Clinical translation is a multifaceted process that involves not only assessing safety and efficacy but also addressing regulatory and ethical considerations. Researchers must navigate the complex landscape of clinical trial design, patient recruitment, and regulatory approvals. Additionally, they must establish standardized protocols for H_2_ administration, dosing, and monitoring to ensure consistency and reliability across clinical trials.

In conclusion, the pursuit of H_2_-primed MSC therapies holds immense promise but is accompanied by intricate challenges. Achieving the ideal dosage, ensuring long-term safety, and successfully transitioning from preclinical research to large-scale clinical trials are all critical aspects of this journey. Addressing these complexities through rigorous research and collaboration is essential to realize the full potential of H_2_-primed MSC therapies and offer innovative solutions for a wide range of medical conditions.

## 7. Conclusions

In the dynamic landscape of regenerative medicine and immunotherapy, the priming of MSCs with molecular hydrogen (H_2_) presents a groundbreaking approach with the potential to revolutionize medical interventions. This innovative strategy has unveiled a spectrum of exciting applications across diverse medical domains, offering a glimmer of hope for patients with various debilitating conditions. However, the realization of this therapeutic promise hinges on the meticulous navigation of several intricate challenges and considerations.

One of the foremost challenges is the quest for the optimal dosage—a precise calibration of H_2_ concentration and duration of exposure necessary for effective MSC priming. This intricate task remains an ongoing area of research, demanding continuous exploration to strike the right balance for maximal therapeutic impact. Achieving this balance involves considering a myriad of factors, including the specific disease or condition being targeted, individual patient variations, and the most suitable administration method. Researchers must remain vigilant in their pursuit of the ideal dosing regimen to harness the full potential of this innovative therapy.

Safety and long-term effects are equally paramount considerations in the journey of implementing H_2_-primed MSC therapies. While H_2_ is generally considered safe, comprehensive investigations are imperative to unveil any potential interactions with concurrent medications or therapies and to ensure the sustained safety profile of H_2_ administration. Safety assessments must extend beyond short-term exposure, encompassing long-term monitoring to detect any unforeseen adverse effects or interactions that may arise during extended therapy periods.

The translation of promising laboratory findings into tangible patient care poses a significant hurdle. Rigorous clinical trials are not merely desirable but essential to ascertain the actual safety, efficacy, and clinical utility of H_2_-primed MSC therapies. This transition from bench to bedside is pivotal in realizing the potential of these innovative therapies to address a broad spectrum of medical conditions, offering renewed hope to patients on a global scale. The clinical trials must adhere to rigorous protocols, account for ethical considerations, and encompass diverse patient populations to provide comprehensive insights into the therapy’s effectiveness.

In conclusion, the integration of molecular hydrogen with MSCs signifies a promising advancement in regenerative medicine and immunotherapy, presenting innovative solutions for challenging medical conditions. The journey to harness the full potential of H_2_-primed MSC therapies involves critical considerations, including optimal dosage calibration, safety assessments, and the translation of laboratory findings into rigorous clinical trials. The continuation of meticulous research, collaborative efforts, and robust clinical investigations is paramount to realizing the transformative impact of this approach on global patient care. H_2_-primed MSC therapies hold significant promise for the future, offering novel avenues for healing and enhanced quality of life.
